# Ultra-Processed Foods, Diet Quality and Human Health

**DOI:** 10.3390/nu15132890

**Published:** 2023-06-26

**Authors:** Monica Dinu, Daniela Martini

**Affiliations:** 1 Department of Experimental and Clinical Medicine, University of Florence, 50134 Florence, Italy; 2 Department of Food, Environmental and Nutritional Sciences (DeFENS), University of Milan, 20122 Milan, Italy

The increase in the volume of industrially processed products in the global food supply has coincided with an increasing prevalence of obesity and non-communicable diseases in many countries, suggesting that ultra-processed foods (UPF) consumption may be detrimental to human health. However, studies are still limited and underline the need to better understand the main determinants of their consumption and the mechanisms that may explain the associations between these products and human health. The Special Issue “Ultra-Processed Foods, Diet Quality and Human Health” aimed to collect new studies investigating the relationship between the consumption of UPF, diet quality and human health, including those aiming to: (i) develop new tools to better determine the rate of consumption of UPF in the population; (ii) investigate the rate of consumption of UPF in different subgroups of the population, including subjects following different dietary patterns; (iii) analyze the relationship between the consumption of UPF and markers of health status; and (iv) explore possible mechanisms behind associations between the consumption of processed foods and health.

This Special Issue provides a series of 21 contributions, with 18 original articles, 1 narrative review, 1 systematic review, and 1 meta-analysis. Some of the articles were devoted to the analysis of the amount of UPF consumed in different populations and over the years. Romero Ferreiro and colleagues estimated UPF consumption in Spain from 1991 to 2008, finding a 10.8% increase in UPF consumption between 1991 and 2008 [1]. Bertoni Maluf et al. described UPF consumption in adults living in Switzerland, finding a median UPF energy intake of 587 kcal/day (range 364–885), corresponding to 28.7% (range 19.9–38.9) of total energy intake [2]. Both studies found higher UPF intake in young participants and large differences between different geographical areas of countries.

It has been hypothesized that the harmful effects of UPF on human health may be related to the worse diet quality of subjects with higher UPF intake. In this regard, Dinu et al. studied UPF consumption in a group of Italian adults, observing a significant inverse association between adherence to the Mediterranean diet (as assessed by the Medi-Lite score) and the percentage of UPF in the diet [3]. Similar results were found by Tristan Asensi et al. who observed an inverse trend between UPF consumption and adherence to the Mediterranean diet in adults with celiac disease [4]. The association between UPF consumption and diet was also considered by Nansel and colleagues, who found that UPF intake during pregnancy and postpartum was inversely related to 8 of 13 component scores of the 2015 Healthy Eating Index [5]. This suggests that the higher the UPF intake, the lower the diet quality. Finally, analyzing data on 8688 Italians from the Italian Nutrition & Health Survey (INHES), Bonaccio et al. observed that late eaters had higher UPF intake and lower adherence to the Mediterranean diet than early eaters [6]. Overall, these data seem to suggest that a reduction in UPF intake can also be achieved by promoting the Mediterranean diet, adherence to which was correlated with a higher quality of life during the COVID-19 lockdown among Brazilian and Spanish youth aged 3–17 years [7].

Other contributions included in the Special Issue were devoted to exploring the association between UPF consumption and markers of human health or disease risk. In particular, the systematic review of Mambrini et al. [8] evaluated the association between UPF consumption and the incidence of obesity and cardiometabolic risk factors. The analysis of 17 studies showed substantial agreement in defining UPF consumption as being associated with the incident risk of general and abdominal obesity. More limited was the evidence on cardiometabolic risk. The other narrative review [9] summarized the available evidence on the possible relationship between excessive consumption of UPF and low-grade inflammation, focusing on nutritional and non-nutritional components that may explain this relationship. Among different markers of inflammation, Lane and colleagues focused on high-sensitivity C-reactive protein (hsCRP) by analyzing data from the Melbourne Collaborative Cohort Study (MCCS), finding that each 100 g increase in UPF intake was associated with a 4.0% increase in hsCRP concentration [10].

Two studies conducted in China analyzed data from the China Health and Nutrition Survey 1997–2015. In the first study, the authors observed that the higher the UPF consumption, the higher the incident rates of hypertension, with hazard ratios (HRs) for UPF intake of 1–49, 50–99, and ≥100 g/day of 1.00 (95% CI: 0.90–1.12), 1.17 (95% CI: 1.04–1.33), and 1.20 (95% CI: 1.06–1.35), respectively, compared with non-consumers [11]. In the second study, the same authors used the data to assess the association between UPF consumption and diabetes in Chinese adults. The odds ratios (ORs) of diabetes for people with a mean UPF consumption of 1–19, 20–49, and ≥50 g/day were 1.21 (95% CI: 0.98, 1.48), 1.49 (95% CI: 1.19, 1.86), and 1.40 (95% CI: 1.08, 1.80), respectively, compared with non-consumers [12]. Diabetes was also considered in a systematic review with meta-analysis that investigated maternal consumption of UPF and perinatal outcomes [13]. Authors observed that maternal consumption of UPF-rich diets was associated with an increased risk of gestational diabetes mellitus and preeclampsia, highlighting the need to reduce UPF consumption during the gestational period to prevent adverse perinatal outcomes.

Other associations between UPF and detrimental health effects were explored by Koniecza and coworkers who observed that each 10% daily increment in UPF consumption in 1 year was associated with higher levels of biomarkers related to non-alcoholic fatty liver diseases (i.e., non-invasive fatty liver index and hepatic steatosis index) in a cohort of 5867 older participants with overweight/obesity and metabolic syndrome from the PREDIMED-Plus trial, following for 1 year [14]. 

High UPF consumption was also found to be associated with an increased risk of depression and depressive symptoms. In a sample of 596 young Italian adults, Godos and colleagues showed that individuals in the highest quartile of UPF consumption were more likely to have depressive symptoms [15]. As this association became stronger when adjusted for other confounding factors, including adherence to the Mediterranean diet as a proxy for diet quality, the authors suggest that dietary components other than nutritional quality may play a role in the reported association. Similar results were observed in the Korean population of The Korea National Health and Nutrition Examination Survey, where 9463 subjects were analyzed [16]. However, in a sex-specific stratification, only women showed a significant association between higher UPF consumption and depression.

The other studies published in this Special Issue focused on different aspects, such as the overlap between the NOVA classification and other systems. In this regard, Angelino and colleagues compared the level of processing (assessed by NOVA) and nutritional quality (assessed by nutritional values, the Nutri-Score and the NutrInform battery) of breakfast cereals available on the Italian market and found a partial overlap between the NOVA classification and the systems based on the nutritional quality of foods [17]. Similarly, Grech et al. used data from the 2011–2012 National Nutrition and Physical Activity Survey, a large cross-sectional study representative of the Australian population, to compare the NOVA classification system with the Australian Dietary Guidelines (ADG) in classifying foods as healthy and unhealthy through their effectiveness in predicting energy overconsumption and body mass index (BMI) [18]. The analysis demonstrated considerable overlap between the NOVA and ADG classification systems, but some discrepancy emerged between the system that best identifies foods to avoid in Australia, with many culinary ingredients classified as unhealthy in ADG and most international dietary guidelines, but not in NOVA.

Finally, Krois and colleagues focused on dietitians’ knowledge of the NOVA food classification system and their attitudes toward the classification of products containing whole grains [19], while Camargo et al. conducted a descriptive and exploratory analysis of the healthiness of 823 culinary recipes shared during a 6-month period on popular Brazilian YouTube^®^ cooking channels, considering the degree of ingredient processing [20]. Only one study was conducted in animal models, analyzing the effects of Zn supplementation on the gut microbiota, intestinal barrier, and blood–brain barrier in Wistar rats fed a cafeteria diet (CAF) rich in UPF [21]. The results showed that chronic consumption of CAF causes dysbiosis, morphological changes and decreased levels of SCFA in the colon, as well as increased saturated fatty acids.

The Guest Editors would like to thank all the authors, the reviewers who contributed to the success of this Special Issue, and the *Nutrients* team for their valuable and constant support. By providing up-to-date assessments of UPF consumption and health implications, but at the same time highlighting some criticisms about the overlap of NOVA classification with other types of information provided to the consumers, these reports support the importance of providing new research on this topic. In particular, a better elucidation of the mechanisms through these products may exert a detrimental effect on human health and evidence from intervention studies seem to be crucial to better understand if future public health nutrition policies are needed.
